# Identification of nicotine-seeking and avoiding larval zebrafish using a new three-choice behavioral assay

**DOI:** 10.3389/fnmol.2023.1112927

**Published:** 2023-03-30

**Authors:** Henning Schneider, Anna Pearson, Drew Harris, Sabrina Krause, Andrew Tucker, Kaitlyn Gardner, Kuzivakwashe Chinyanya

**Affiliations:** Department of Biology, DePauw University, Greencastle, IN, United States

**Keywords:** addiction, self-administration, drug-use, varenicline, smoking cessation

## Abstract

**Introduction:**

Nicotine dependence is one of the main causes of preventable diseases in the United States. Nicotine-seeking and avoidance behavioral assays in larval zebrafish could be used for identifying potential new pharmacotherapeutics in an early phase of drug discovery and could facilitate the identification of genes and genomic variations associated with nicotine-seeking and avoidance behavior.

**Methods:**

A new three-choice behavioral assay has been developed for the identification of nicotine-seeking and avoiding larval zebrafish. The three choices are represented by three compartments of a gradient maze. Video-recording and subsequent quantitative analysis of the swimming track was carried out using EthovisionXT (Noldus).

**Results:**

Three behavioral phenotypes could be identified. Nicotine-seeking larval zebrafish occupied nicotine compartments for longer periods and entered the nicotine-containing compartments most frequently. Nicotine-avoiders spent most of the cumulative time in the water compartment or entered the water compartment most frequently. Non-seekers remained in the center compartment for most of the time. In the gradient maze, about 20–30% of larval zebrafish had a preference for low nicotine concentrations whereas nicotine avoidance was stronger at higher nicotine concentrations. Lower concentrations of nicotine (0.63 μM, 6.3 μM) resulted in higher percentages of nicotine seekers whereas high nicotine concentrations (63 μM, 630 µM) resulted in higher percentages of nicotine avoiders. Pre-treatment of larval zebrafish with nicotine slightly increased the percentage of nicotine avoiders at lower nicotine concentrations. Treatment with varenicline strongly increased the percentage of nicotine avoiders at lower nicotine concentrations.

**Conclusion:**

The results show that larval zebrafish have individual preferences for nicotine that could change with drug treatment. The three-choice gradient maze assay for larval zebrafish provides a new testing paradigm for studying the molecular and cellular mechanisms of nicotine action and the discovery of potential new pharmacotherapeutics for the treatment of smoking cessation.

## 1. Introduction

The use of nicotine products remains one of the leading causes of preventable diseases in the United States.^1^ Cigarette smoking leads to diseases such as lung cancer, heart disease, and COPD. Although smoking tobacco products in the United States has declined from 20.9% in 2005 to 12.5% in 2020, about 30.8 million adults, in the United States still smoke or regularly use some form of nicotine product. Adults, 45–64 years old, represent the age group with the highest percentage of smokers (14.9%). The rate of smoking is the highest among non-Hispanic American Indian/Alaska native adults (27.1%) and the highest in adults with an annual household income of less than $35,000 (20.2%). Youth use of tobacco product in the US is at 4.5% for middle school students and 16.5% for high school students (Park-Lee et al., 2022). Vaping liquids used in Juul’s, and e-cigarettes contain nicotine and are popular among teenagers. Because the success rate of quitting smoking is low as 75% of individuals who quit smoking relapse within 3 months and 90% within 12 months, additional treatments and therapies are needed to improve the rate of smoking cessation (Ebbert et al., 2010; Jordan and Zhen-Xiong, 2018; Hurt et al., 2022). The use of nicotine products varies between individuals. Most nicotine users experience nicotine in adolescence for the first time. Transitions to avoidance (no nicotine use), controlled nicotine use and nicotine dependence (uncontrolled use) depend on both genetic and environmental factors (Wang et al., 2021).

While rodents are the standard model for studying the actions of drugs of addiction such as nicotine and for discovering potential new chemicals for nicotine cessation treatments, zebrafish (*Danio rerio*) have emerged as an alternative model (Pan et al., 2012; Parker et al., 2013; Kaleuff et al., 2014). Nicotine induces robust acute behavioral changes in all post-embryonic stages of zebrafish as well as nicotine-induced long term behavioral changes in adult zebrafish (Petzold et al., 2009; Brennan et al., 2011). The high fecundity and the fast development of zebrafish to the larval stage allows the screening of nicotine-seeking behavior as early as 5 days after fertilization (5 dpf) (Schneider, 2017). The genetic toolbox for analyzing and modifying the zebrafish genome is enormous and includes a sequenced reference genome and methods for genome modifications such as CRISPR and TALENs (Blackburn et al., 2013; Ata et al., 2016). In addition, the small size of larval zebrafish facilitates behavioral testing and fast screening of large number of chemicals (high throughput) (Rihel et al., 2010; MacRae and Peterson, 2015; Brady et al., 2016; Lee et al., 2022). While the behavioral response of larval zebrafish to acute exposures to nicotine (acute nicotine response, ANR) is routinely used in drug-development projects, they do not allow larval zebrafish to titrate their exposure to nicotine as in self-administration tests in rodents. Behavioral choice tests for drugs of dependence are less developed in larval zebrafish compared to adult zebrafish (Schneider, 2017; Nathan et al., 2022).

The initial experience associated with nicotine use impacts the future user patterns of nicotine products such as avoidance, casual use and dependence of nicotine products (US Department of Health and Human Services, 2014). To better understand the underlying mechanisms of nicotine use, behavioral choice tests in which animals can self-administer drugs are more suitable (Perkins, 1999). The self-administration experiments for drugs of abuse in rodents have been essential for the discovery of neuronal mechanisms regulating nicotine use but are more difficult to conduct in zebrafish and especially in larval zebrafish (Krishnan et al., 2014; Bosse and Peterson, 2017; Bosse et al., 2021a,b; Gallois et al., 2022; Nathan et al., 2022). Based on mazes that have been successfully used for rodents and single-chamber behavioral choice tests for larval zebrafish, we developed a three-compartment gradient-maze for measuring nicotine-seeking and avoidance behavior of individual larval zebrafish. Because nicotine can be taken up into the body through the skin, freely swimming larvae can self-administer or titrate nicotine by choosing to stay in the nicotine, a center, and a water compartment. The gradient maze is designed to allow the selective delivery and exposure to drugs as larval zebrafish can move freely between compartments. In this study, nicotine seeking and avoidance behavior were tested in the gradient maze at different nicotine concentrations, after nicotine pre-treatment and treatment with the nicotinic acetylcholine receptor agonist varenicline, the active drug in the smoking cessation drug Chantix (Coe et al., 2005; Benowitz, 2009; Rigotti et al., 2022).

## 2. Methods

### 2.1. Zebrafish

Adult wildtype zebrafish (PWT) were obtained from a local supplier and maintained at 28–29 degrees Celsius on a 14-h light/ 10-h dark cycle at the zebrafish facility of DePauw University. Embryos were collected from breeding tanks and placed in 100 mm Petri dishes filled with 25 ml half-strength (0.5x) embryo water (15 mM NaCl, 0.5 mM KCl, 1.0 mM MgSO_4_, 0.15 mM KH_2_PO_4_, 0.05 mM Na_2_HPO_4_, 1.0 mM CaCl_2_ and 0.7 mM NaHCO_3_; after Brand et al., 2002). Larval zebrafish were raised in Petri dishes in embryo water at 28–29 degrees Celsius on a 14-h light/10-h dark cycle on LED light boxes in an incubator or behavioral room. Experiments were carried out with larval zebrafish on days 6–8 post fertilization (6–8 dpf). All protocols involving zebrafish had been approved by IACUC at DePauw University.

### 2.2. Chemicals

Working concentrations of nicotine solutions were prepared by serial dilution of a 10 M L-nicotine stock solution (Acros, AC181420250) using embryo water. Varenicline tartrate (Tocris, #3754) was obtained in powder-form and dissolved in embryo water to obtain a 10^−2^ M stock solution. Subsequent serial dilutions were carried out as needed with embryo water to obtain the desired working concentrations.

### 2.3. Mazes

**Figure 1:**
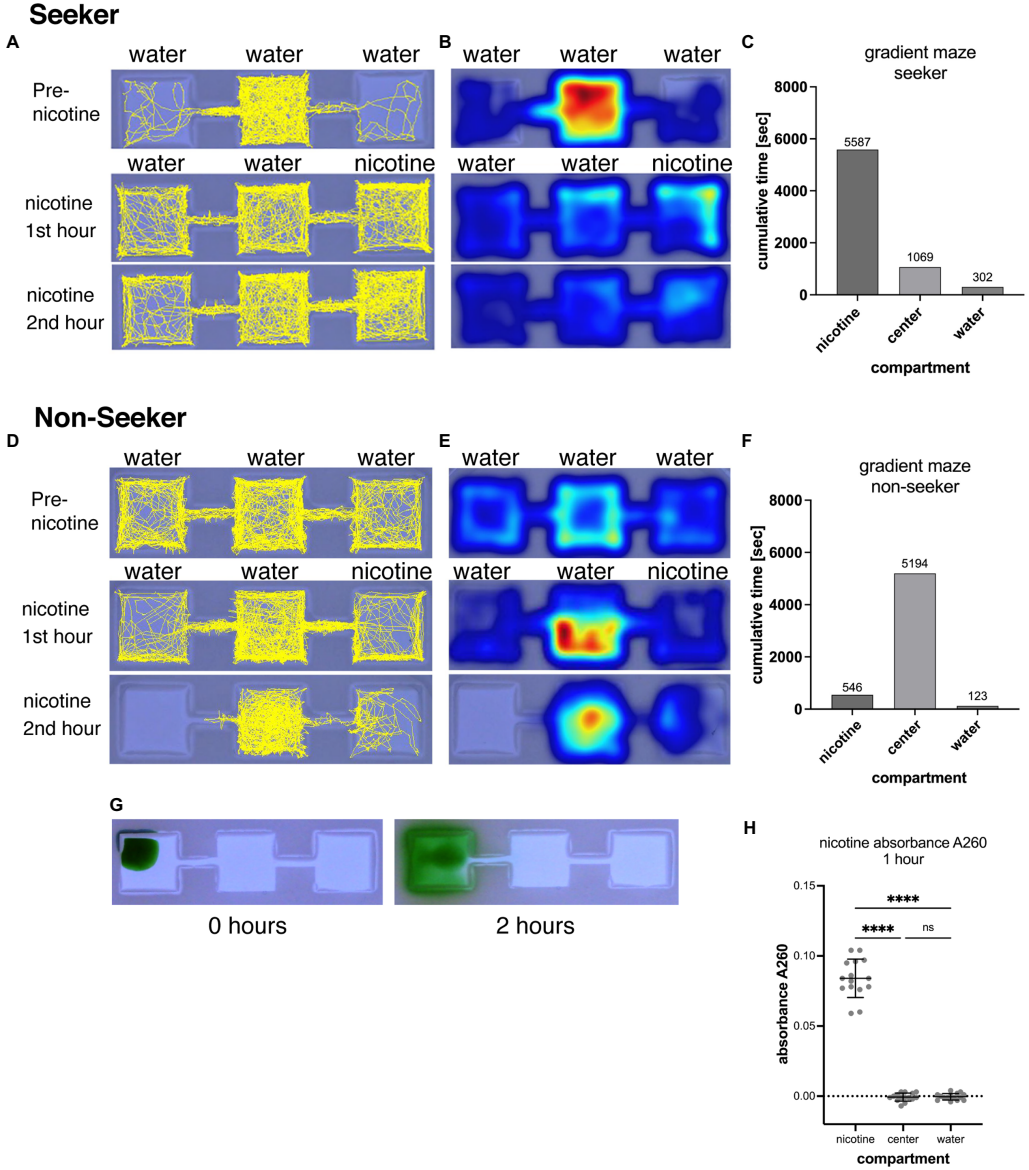
Shows the representation of swimming activities of a nicotine seeker **(A–C)** and a non-seeker **(D–F)**. Tracks of larval zebrafish (yellow) as generated by EthovisionXT are shown in **(A)** for a seeker and **(D)** for a non-seeker. Heatmaps of the corresponding experiments are shown in **(B)** for the seeker and **(E)** for the non-seeker. Warmer colors in heatmaps indicate more time spent in a location. Examples of the analysis for a single larval zebrafish are shown in **(C)** for the seeker and in **(F)** for the non-seeker. **(G)** Shows the diffusion of 5 μl green food dye added to the left outer compartment of a gradient maze. After 2 h the dye is mostly contained in the left compartment. **(H)** Absorbance measurements (at 260 nm) of water samples (25 μl) taken from the nicotine, center and water compartments of 15 gradient mazes 1 h after pipetting 5 μl of 200 mM nicotine solution into the nicotine compartments. Each data point represents one measurement from one compartment. Absorbance could be detected in nicotine compartments but not in center and water compartment. Nicotine compartment: mean absorbance 0.084 (SD = 0.0137; *n* = 15); Center compartment: mean absorbance −0.0007 (SD = 0.002895; *n* = 15); Water compartments: mean absorbance −0.0004 (SD = 0.002324; *n* = 15). ****p < 0.0001; ns > 0.9999.

Gradient mazes were made with 1.5% agarose dissolved in boiling embryo water and reusable 3D-printed gradient maze molds. The molds were designed using AutoCAD (Autodesk) (Supplementary Figure S1). The area of each compartment was 15 mm × 15 mm. The connectors between compartments were 10 mm long and 2 mm wide. These dimensions were selected after testing diffusion of chemicals and movement of larval zebrafish. The connectors were narrow enough to reduce the diffusion of directly added chemicals out of the compartment and wide enough for larval zebrafish to pass through and move between compartments. A center compartment was connected on two opposite sides to outer compartments (Figure 1). The height of molds was 30 mm to allow easy removal. Either five 3D-printed molds [polylactic acid (PLA) material] were positioned evenly spaced in a single one-well plate^2^ (#229501) or three 3D-printed molds were placed into 100 mm polystyrene Petri dishes (VWR #25384-342). Agarose was added to embryo water and completely dissolved by boiling in a microwave oven. After cooling for 10 min at room temperature, 55 ml of agarose solution was poured into the dish containing the molds and allowed to solidify for at least 45 min at room temperature. Molds were removed and any thin layer of solidified agarose that had formed underneath the molds at the bottom of the mazes was carefully removed using a plastic pipette tip. Depth of mazes was 8–9 mm. Mazes were covered with the lid, sealed with parafilm and stored for up to 3 days. For experiments, each gradient maze was filled with 5.0 ml embryo water to a water level of 7–8 mm measured in the center of each compartment.

### 2.4. Behavioral experiments

A single behavioral setup consisted of a video-camera (Canon Vixia HF R80 and R82) mounted 24 inches above an LED – light box (Displays2go.com; #APFLP1117) on a black wooden frame (Supplementary Figure S2). One one-well plate with five gradients mazes or one Petri dish with three gradient mazes was placed onto the LED light box and centered in the viewfinder of the camera. White artboard shielding was placed to surround the plate or dish to block any visual stimulation that could interfere with the free movement of larval zebrafish in the gradient maze. The shielding was temporarily removed for pipetting nicotine into the gradient mazes and replaced after pipetting was completed. Experimenters left the behavioral lab during the video-recording periods. To scale up the number of larval zebrafish that could be tested in one experiment session, we constructed three behavioral setups and placed them next to each other onto the same bench in the behavioral lab. Three one-well plates with 15 larval zebrafish or three Petri-dishes with 9 larval zebrafish total were tested in each experiment session.

Because larval zebrafish are sensitive to the environmental and water temperature all experiments were performed with temperature equilibrated mazes, solutions, and plasticware in a dedicated behavioral lab in which the room temperature was maintained between 27 and 29 degrees Celsius. Mazes and embryo water brought in from outside the behavioral lab were temperature equilibrated for at least 24 h before use in the behavioral lab.

Zebrafish embryos that were obtained from breeding setups using standard procedures, were raised in 100 mm polystyrene Petri dishes filled with 25 ml embryo water to the larval developmental stage in a 28 degree Celsius incubator on a 14 h light/10 h dark cycle and tested on day 6–8 after fertilization (6–8 dpf). Petri dishes contained 25–30 embryos. On the day of the experiment, Petri dishes with larval zebrafish were transferred from the incubator onto the LED light box of the video recording setup in the behavioral lab, the lid of the dish was removed, and the larval zebrafish could temperature and light adapt for 60 min. Each temperature-equilibrated maze was filled with 5 ml temperature-equilibrated embryo water using temperature equilibrated serological pipettes. One larval zebrafish (6–8 dpf) was transferred into the center compartment of one gradient maze each in the dish or plate using temperature equilibrated plastic transfer pipettes. The dish or plate with gradient mazes was positioned in the viewfinder of a video camera and the focus was adjusted manually. Movement activity was recorded for 30 min before nicotine solution was added into one compartment (pre-nicotine phase). After the 30-min pre-nicotine phase, videorecording was stopped and 5 μl nicotine solution was pipetted into one outer compartment of each maze. White artboard shielding was always placed around the plate or dish before videorecording was started (Supplementary Figure S2). The calculated dilution factor of nicotine solution was 315 in the nicotine compartment. The 5 μl of a 200 mM nicotine solution pipetted into the nicotine compartment, would be diluted 315 times based on the surface area of each compartment (15 mm × 15 mm) and the height of the water level in the center of compartments (~7 mm) resulting in a concentration of approximately 635 μM. The 5 μl nicotine solution was directly pipetted into a lower corner of one outer compartment (Figure 1 and Supplementary Movie S3). Pipetting the nicotine solution into the lower corner of the compartment is critical for preventing diffusion of nicotine into the center compartment. To avoid adding nicotine directly onto larval zebrafish, any larval zebrafish in an outer compartment were transferred to the center compartment immediately before adding nicotine. The white artboard shielding was placed to surround the plates with the mazes and videorecording was turned on. After 2 h, videorecording was stopped and larval zebrafish were transferred into 6-well plates, one larval zebrafish per well, each filled with 5 ml embryo water. Larvae were transferred and raised in the zebrafish facility. After one-week, larval zebrafish were transferred to larger containers and ultimately into 1.4 l zebrafish tanks (Aquatic Ecosystem, Aquaneering) kept on a zebrafish tank system (AquaticEcosystems).

### 2.5. Nicotine pre-treatment and varenicline treatment of larval zebrafish

For nicotine pre-treatments, larval zebrafish were first placed on the day of the experiment into 100 mm Polystyrene Petri dishes filled with 25 ml nicotine solution (1 μM) and kept on the LED light panel of the behavioral setup for 1 h. Then, larval zebrafish were transferred to mazes for the 30-min pre-nicotine equilibration on the light panel (no nicotine exposure in equilibration period) and videorecorded after placing white artboard shielding around the plate or dishes. Then nicotine solution was added to the mazes as described above, and videorecording resumed for 2 h. For varenicline testing, gradient mazes were filled with 5 ml embryo water containing 20 μM varenicline tartrate, all temperature equilibrated and placed onto the light panel. Then larval zebrafish were transferred into the center compartment of the mazes containing varenicline and incubated and video-recorded for 1 h. After the 1-h equilibration period and varenicline treatment, nicotine was added to the mazes as described above. Videorecording was carried out during the 1 h of varenicline incubation period before addition of nicotine to compartment and the 2 h after pipetting of nicotine into the mazes. Both, varenicline and nicotine were present in the mazes over the 2-h video recording period. For comparison, one experimental group of larval zebrafish was not treated with varenicline before addition of nicotine to nicotine compartments. Experiments without varenicline treatment and without the addition of water instead of nicotine served as additional control.

### 2.6. Dose dependency tests

When testing the nicotine seeking and avoidance behavior at different nicotine concentrations, experimental conditions were randomized. In one test session, three one-well plates with five gradient mazes each were setup and used simultaneously – one plate on each of three behavioral recording setups. Using this arrangement, three different nicotine concentrations were tested simultaneously on larval zebrafish from the same clutch. Each one of the three plates was set up with a different nicotine concentration or water (as control). In addition, the outer compartment into which the 5 μl nicotine solution (or water for controls) was pipetted varied between plates. Nicotine or water was pipetted into the left or right outer compartment. The randomization was carried out to account for potential clutch-specific behavioral variations.

### 2.7. Repeated testing

For repeated testing, individual larval zebrafish were tested on day 6, 7, and 8 post fertilization (dpf) using the same testing paradigm as described for behavioral testing above. After the first gradient maze test on 6 dpf, larval zebrafish were transferred to 6-well plates, one larval zebrafish per well, and kept in an incubator at 28 degrees Celsius and a 14 h light/10 h dark cycle until the testing on the second day. For the second test at 7 dpf, larval zebrafish were transferred from 6-well plates into new gradient mazes filled with embryo water. Behavioral experiments followed the same protocol on the first test day an as described above including the 30-min equilibration period (pre-nicotine) and a 2-h videorecording after nicotine had been added to one outer compartment. After this second day test, larval zebrafish were transferred back into the 6-well plates which were placed into the incubator under described temperature and light conditions. For the recording on the third day at 8 dpf, we followed again the same procedure as on the first 2 days of testing. Each larval zebrafish was tested on day 6, 7, and 8 post fertilization (pdf). For each experiment, 5 μl of 200 μM nicotine was added to the nicotine compartment as described above. For the statistical analysis, we used a two way ANOVA test to account for the different days of testing and the different compartments.

### 2.8. Data analysis and statistical test

Files of video recordings of experiments were imported into EthoVisionXT 15 (Noldus) for acquisition and subsequent analysis following the manufacturer’s instructions. The acquisition parameters (arena and detection settings) were selected so that larval zebrafish were tracked continuously. To avoid that missing tracking coordinates introduced errors, especially for the analysis of entrances into compartments, we used the track editing tool in EthovisionXT. Swimming tracks were examined for accuracy of tracking quality (no tracks outside of a gradient maze). The swimming tracks of larval zebrafish were analyzed for the cumulative duration spent in compartments of mazes and the frequency of entering the nicotine compartment. Acquired data were exported to Excel (v16, Microsoft) for grouping results from all mazes in one plate or dish. The data were imported to Prism9 (Graphpad) that we used for graphing and statistical analyses including calculation of means and standard error means (±SEM). One-way ANOVA tests (Kruskal–Wallis with Dunn’s posttest) were used for the analysis of cumulative duration and frequency of entering compartments in behavioral tests for the screening of nicotine seekers and avoiders, dose-dependency of nicotine seeking and avoidance behavior, nicotine pre-treatment experiments and varenicline experiments. A two-way ANOVA test was used for the analysis of repeated testing experiments.

## 3. Results

### 3.1. Nicotine-seeking in the gradient-maze for identification of nicotine-seekers and avoiders

A gradient-maze was developed for direct application of nicotine into the maze and identification of nicotine seekers and avoiders. A center compartment was linked by a narrow connector to outer compartments, one on each side (Figure 1). In experiments, nicotine was added to one outer compartment (nicotine compartment) while the other outer compartment opposite to the nicotine-compartment did not receive nicotine (water compartment). Following a 30-min acclimation period that started immediately after placing a single larval zebrafish into the central compartment, a nicotine solution (5 μl, 200-times concentrated) was added to one of the outer compartments. Nicotine-seeking larval zebrafish were defined by the cumulative time and number of entrances (frequency) into the nicotine compartment over the 2-h observation period. Larval zebrafish with the highest cumulative time in the nicotine compartment were identified as duration seekers (Figures 1A–C). Larval zebrafish with the longest cumulative duration spent in the water compartment were identified as duration avoiders. Larval zebrafish that entered the nicotine compartment most frequently compared to the center and water compartment were identified as frequency seekers. Larval zebrafish that entered the water compartment most frequently were identified as frequency avoiders. Larval zebrafish that spent most of the time in the center compartment or entered the center compartment most frequently were identified as non-seekers. (Figures 1D–F). The example of the track map in Figure 1A shows a nicotine seeker that spent most of the time in the center compartment before nicotine application but shifted toward the nicotine compartment after nicotine was added to the maze. Corresponding heat maps (Figure 1B) provided information about the activity pattern and were used only to determine if a larval zebrafish had stationary phases during which they a did not move. The example of a non-seeker (Figures 1D,E) showed the shifted away from the nicotine compartment once nicotine was added as well as shifting away from the water compartment and became more stationary in the center compartment (Figure 1E). A significant shifting of larval zebrafish between compartments after application of nicotine was used to determine responses to nicotine and drug treatments.

The volume and concentration of nicotine solution that was pipetted into the nicotine compartment was selected to limit diffusion of nicotine out of the compartment and generating a concentration around 1 μM in the nicotine compartment. Larval zebrafish had a 100% 24 h-survival rate at 1 μM nicotine and seemed to be attracted by nicotine (Krishnan et al., 2014). Indeed, the narrow link between the center compartment suppressed the diffusion of added liquids from the outer (nicotine) to the center compartment over the 2-h video-recording phase as shown (Figure 1G). When 5 μl of concentrated food dye was pipetted into an outer compartment, only a small amount of liquid could diffuse into the center compartment, and none into other outer compartment which did not appear to contain any food dye after 2 h. To determine the concentration and potential diffusion of nicotine in the gradient maze we measured the absorbance of nicotine at 260 nm (Willits et al., 1950). Solutions were sampled from all three compartments after the first hour of adding 5 μl of nicotine (200 mM) to the nicotine compartment when the gradient maze was filled with 5.0 ml embryo water. After 1 h mean positive absorbance of 0.084 (SD = 0.0137; *n* = 15) could be detected in the nicotine but not in the center (mean = −0.0007; SD = 0.002895; *n* = 15) and water compartments (mean = −0.0004; SD = 0.002324; *n* = 15) indicating that nicotine did not diffuse from the nicotine compartment into the center compartment (Figure 1F). However, the calibration measurements for this spectrophotometric nicotine measurement showed that absorbances of concentrations below 10 μM could not be detected reliably because of negative absorbances. Based on the A260 values, adding a 5 μl volume of nicotine (200 mM) resulted in a mean concentration of 630 μM (using the obtained standard curve *y* = 0.1633x-0.019; *n* = 15) in the nicotine compartments after 1 h of adding nicotine. Based on these results we concluded that adding 5 μl of 200 mM nicotine to the compartment would results in a concentration of 630 μM nicotine, 5 μl of 20 mM nicotine in a nicotine concentration of 63 μM, 5 μl of 2 mM nicotine in a nicotine concentration of 6.3 μM and 5 μl of 200 μM in a nicotine concentration of 0.63 μM. The design of the gradient maze allowed easy delivery and retention of nicotine in compartments in which larval zebrafish can swim freely following their preferences.

### 3.2. Identification of nicotine-seekers and avoiders

**Figure 2:**
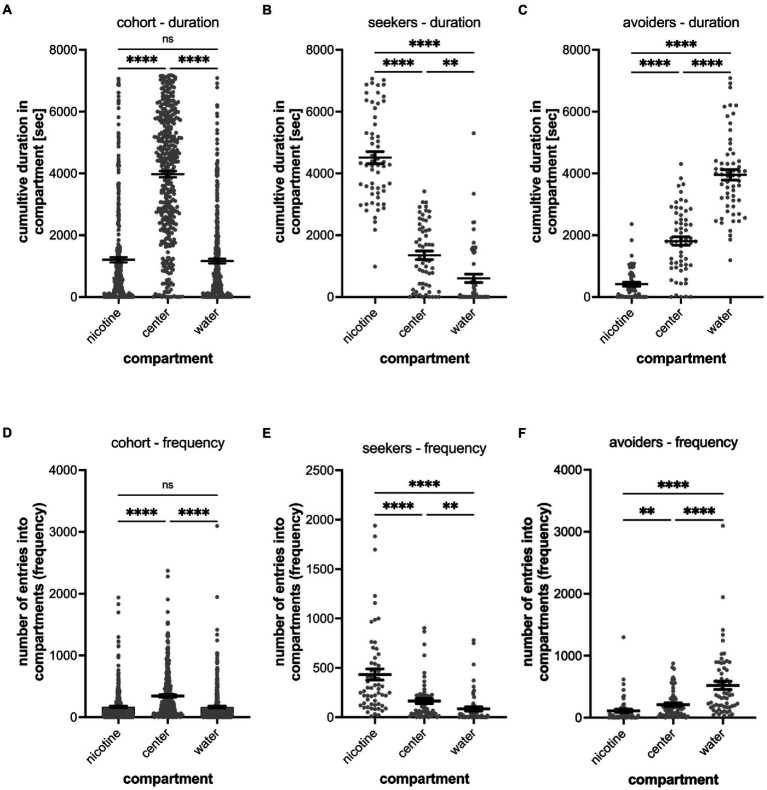
Shows the quantitative analysis of the cumulative duration spent in compartments of the gradient maze **(A–C)** and the number of entrances into compartments (frequency) **(D–F)**. The results for the entire cohort are shown in **(A,D)**, for nicotine seekers in **(B,E)** and for nicotine avoiders in **(C,F)**. Each graph shows the individual datapoints for the nicotine compartment (nicotine), center compartment (center), and the water compartment (water). Data are shown as mean with ±SEM. Statistical significance was tested using a one-way ANOVA Kruskal–Wallis test with Dunn’s comparison test. The results for the cumulative duration of the cohort **(A)** and for the frequency of entering a compartment for the cohort (*n* = 390) establish the behavioral profile baseline for the nicotine-seeker and avoidance test. Nicotine-seekers spent significantly more time in the center compartment compared to the nicotine and water compartment (nicotine vs. center *p* < 0.0001; nicotine vs. water *p* ≥ 0.9999, center vs. water *p* < 0.0001). In **(B)** the behavioral profile of nicotine duration seekers (*n* = 58) has shifted to the nicotine compartment (nicotine vs. center: *p* < 0.0001; nicotine vs. water: *p* < 0.0001, center vs. water: *p* = 0.0046). For nicotine duration avoiders (*n* = 60) in **(C)** the behavioral profile has shifted to the water compartment. **(D)** Nicotine vs. center: *p* < 0.0001; nicotine vs. water: *p* < 0.0001, center vs. water: *p* < 0.0001). The results for entering the nicotine, center and water compartments of the cohort (*n* = 390) are similar compared to the duration profile (nicotine vs. center: *p* < 0.0001; nicotine vs. water: *p* = 0.7969, center vs. water: *p* = 0.0026). In **(E)** the behavioral profile of frequency seekers has shifted to the nicotine compartment (nicotine vs. center: *p* < 0.0001; nicotine vs. water: *p* < 0.0001, center vs. water: *p* = 0.0018). In **(F)** the behavioral profile for frequency avoiders has shifted to the water compartment (nicotine vs. center: *p* = 0.0021; nicotine vs. water: *p* < 0.0001, center vs. water: *p* < 0.0001). Data are shown as mean with ±SEM. Statistical significance was tested using a one-way ANOVA Kruskal–Wallis test with Dunn’s comparison test. ****p < 0.0001; **p < 0.0056; *p < 0.046; ns > 0.9999.

The first series of experiment established behavioral phenotype profiles. The preferences for nicotine were tested on 390 larval zebrafish and a nicotine concentration of 0.63 μM in the nicotine compartment (Figure 2). The cumulative time and the frequency of entering the compartment was analyzed. In gradient maze tests (*n* = 390), larval zebrafish spent most of the time (3,974 s; SEM ± 101.6) in the center compartment (Figure 2A). The nicotine compartment and the water compartment were occupied for similar average cumulative times (nicotine: 1204 s; SEM ± 81.75; water: 1162 s; SEM ± 73.44). The difference between the time spent in the nicotine and water compartment was not significantly different (*p* > 0.9999).

When analyzing the preference of all tested larval zebrafish (cohort), 22.8% or 89 out of 390 tested larval zebrafish were identified as nicotine-seekers at 0.63 μM nicotine. These 89 seekers included 36 super seekers (spent most time in the nicotine compartment and entered the nicotine compartment most frequently), 22 duration seekers (spent most time in the nicotine compartment) and 31 frequency seekers (entered the nicotine compartment most frequently) (Table 1).

**Table 1:** Behavioral Phenotypes in a Cohort of 390 larval zebrafish (100%).

Behavioral Phenotype	Total	%
All Seekers	89	22.8
Duration Seeker	22	5.6
Frequency Seekers	31	7.9
Super Seekers	36	9.2
Duration + Super Seekers	58	14.9
All Avoiders	93	23.8
Duration Avoiders	31	7.9
Frequency Avoiders	26	6.7
Super Avoiders	36	9.2
Duration + Super Avoiders	67	17.2
All Non-seekers	208	53.3

Larval zebrafish with a nicotine duration seeker profile (*n* = 58, duration seekers and super seekers) spent a mean time of 4,513 s (SEM ± 196) in the nicotine compartment, 1,350 s in the center compartment (SEM ± 139) and 605 s (SEM ± 134) in the water compartment (Figure 2B). In comparison, larval zebrafish with a nicotine duration avoider profile (*n* = 67, duration avoiders and super avoiders) spent a mean time of 413 s in the nicotine compartment (SEM ± 65), 1809 s (SEM ± 137) in the center compartment and 3,953 s (SEM ± 170) in the water compartment (Figure 2C). Non-seekers including larval zebrafish that spent most of the time in the center compartment or entered the center compartment most frequently (*n* = 208) made up 53.3% of the tested larval zebrafish in the gradient maze, spent a mean time of 4,945 s (SEM ± 130) in the center compartment, 740 s (SEM ± 61.09) in the nicotine compartment and 651 s (SEM ± 57.81) in the water compartment.

The frequency analysis of larval zebrafish entering the three compartments for the entire cohort (*n* = 390) mirrored the duration results (Figure 2D). The center compartment was entered most frequently (mean 343.9; SEM ± 19.0), while entrances into the nicotine compartment (mean 167.5; SEM ± 13.37) and the water compartment (mean 164.8; SEM ± 15.09) were similar and not significantly different (*p* = 0.1124, *n* = 390). The differences between the number of entrances into the nicotine and center compartments were significantly different (*p* < 0.001); also, the number of entrances into the water compartment compared to the center compartment were significantly different (*p* < 0.001). Larval zebrafish with a frequency seekers profile (*n* = 60, including frequency seekers and super seekers) preferred the nicotine compartment (mean 433 entrances; SEM ± 56) over the center compartment (mean 164 entrances; SEM ± 26) and the water compartment (mean 84 entrances; SEM ± 21) (Figure 2E). The differences recorded for the three compartments varied significantly (nicotine vs. center – *p* < 0.0001; nicotine vs. water – *p* < 0.0001; center vs. water *p* = 0.0018). Larval zebrafish with a nicotine frequency avoider profile (*n* = 62, frequency avoiders and super avoiders) entered the water compartment most frequently (mean 521 entrances; SEM ± 66) compared to the center (mean 211 entrances; SEM ± 29) and nicotine compartments (mean 110 entrances; SEM ± 26) (Figure 2F). The number of entrances between the nicotine and the water compartment (*p* < 0.0001), between the nicotine and the center compartment (*p* = 0.0021) and between the center and the water compartment (*p* < 0.0001) were significantly different.

Out of 390 larval zebrafish that were tested in the gradient maze, 93 (23.8%) were identified as nicotine-avoiders including 36 super avoiders (spent most of the time in the water compartment and entered the water compartment most frequently), 31 duration avoiders (spent most of the time in the water compartment) and 26 frequency avoiders (entered the water compartment most frequently). (Table 1).

Overall, the analysis of cumulative time spent in compartments and the frequency of entering compartments identified behavioral profiles for (1) nicotine-seekers, (2) nicotine-avoiders, and (3) non-seekers. The experimental setup allowed the testing of large numbers of larval zebrafish easily. Environmental and genetic factors associated with behavioral responses to nicotine could potentially be identified by significant shifting of behavioral profiles in test groups.

### 3.3. Dose-dependency of nicotine seeking and avoidance

**Figure 3:**
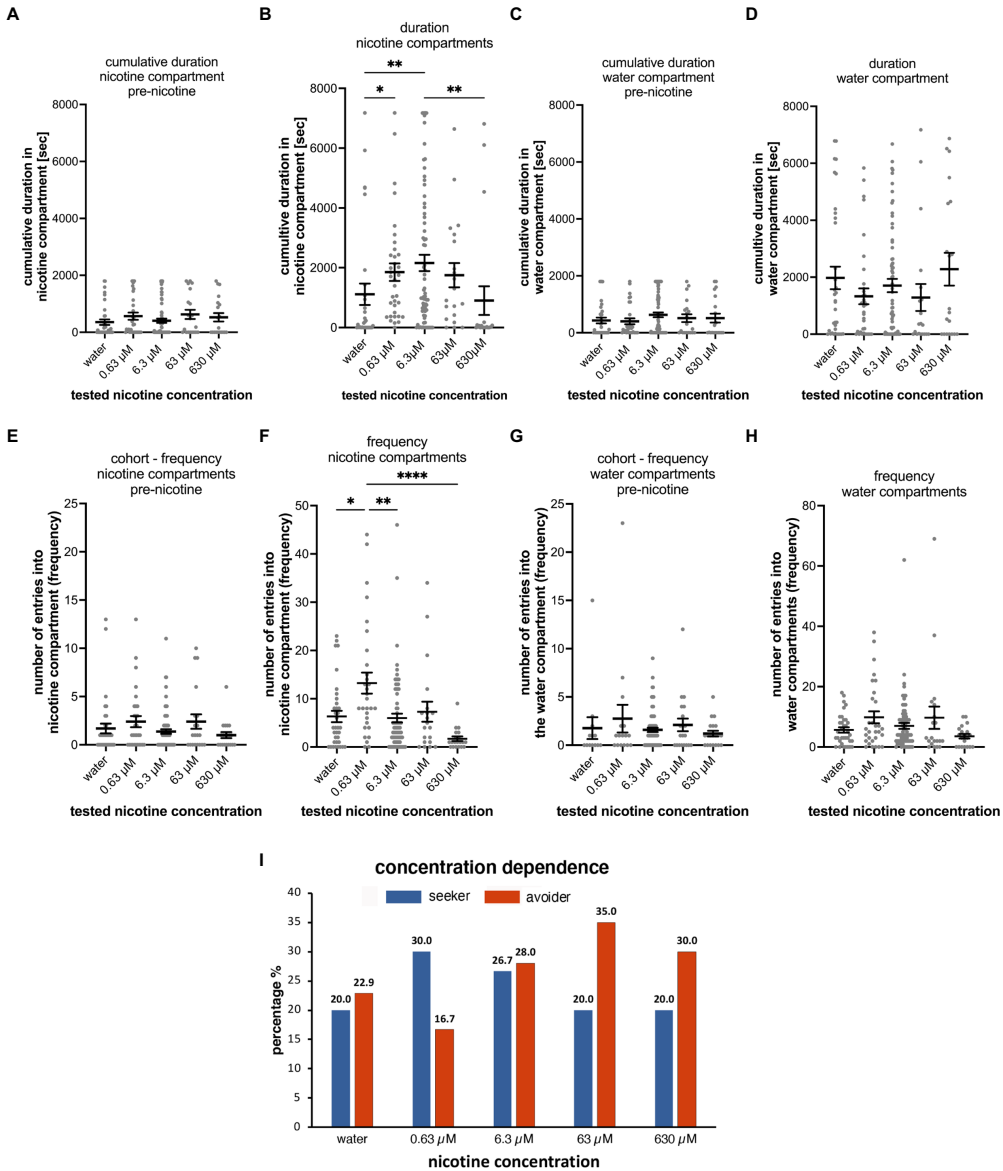
Concentration dependence of nicotine-seeking and avoidance in larval zebrafish. **(A)** Shows the duration spent in the “nicotine” compartment before the addition of nicotine (pre-nicotine). No significant differences (*p* > 0.9999) were detected between water controls (water) and different nicotine concentrations (0.63, 6.3, 63 μM nicotine, and 630 μM nicotine) for the cumulative duration in the pre-nicotine phase of experiments. The duration of the pre-nicotine phase was 30 min compared to the duration of the 2-h nicotine phase after adding nicotine. **(B)** The concentration of nicotine increased the cumulative duration in the nicotine compartment compared to water controls for 0.63 μM nicotine (*p* = 0.0158), and for 6.3 μM nicotine (*p* = 0.0092), but not for 63 μM nicotine (*p* = 0.2627) and 630 μM nicotine (*p* > 0.9999). The difference in cumulative duration in 630 μM nicotine decreased significantly (*p* = 0.0015) compared to the 6.3 μM nicotine concentration. No significant differences in the cumulative duration in the water compartment were apparent in the 30-min pre-nicotine phase **(C)** and the 2-h nicotine phase **(D)** of the experiments. **(E)** The number of entrances into the “nicotine” compartment (frequency) before the addition of nicotine (pre-nicotine) were not significantly different. **(F)** After the addition of nicotine at a final concentration as indicated (0.63, 6.3, 63, and 630 μM) compared to water controls (water) the number of entrances increased in 0.63 μM nicotine (*p* < 0.0001), but not in 6.3 μM nicotine (*p* = 0.7926), 63 μM nicotine (*p* > 0.9999) and 630 μM nicotine (*p* > 0.9999). The number of entrances into the water compartment (frequency) before the addition of nicotine (**G**, pre-nicotine) and after the addition of nicotine **(H)** at tested concentrations were not significantly different. Data are shown as mean ±SEM. Statistical significance was tested using a Kruskal–Wallis test with Dunn’s comparison test. Levels FIGURE 3 (Continued)of significance are indicated. **(I)** The percentage of nicotine-seekers (combined duration seekers, frequency seekers, and super seekers) and avoiders (combined duration avoiders, frequency avoiders and super avoiders) for each tested nicotine concentration (0.63, 6.3, 63, and 630 μM) as well as water controls (water) showed an increase in seekers at 0.63 and 6.3 μM nicotine followed by a decrease in 63 and 630 μM nicotine. Data labels above columns indicate percentages. Most avoiders were counted at 63 and 630 μM nicotine. The lowest percentage of avoiders was found at 0.63 μM nicotine and a slightly higher percentage at 6.3 μM. Individual data points are shown with the mean ±SEM. Statistical significance was tested using a one-way ANOVA Kruskal–Wallis test with Dunn’s comparison test. ****p < 0.0001; **p < 0.0058; *p < 0.049.

To explore whether nicotine concentrations impacted nicotine-seeking and avoidance in larval zebrafish, nicotine concentrations ranging from 0.63 to 630 μM were tested in the gradient maze assay (Figure 3 and Tables 2A,B). Control experiments were carried out in parallel on one of three behavioral setups while nicotine tests were performed on the two additional setups simultaneously with larval zebrafish from the same clutch. In controls, 5 μl embryo water were added to an outer compartment instead of a nicotine solution. For comparison, the movement activity over a 30-min period immediately before the addition of nicotine (pre-nic) to one compartment of the maze was video-recorded and analyzed.

**Table 2A:** Statistical analysis of nicotine dose-dependency - duration in nicotine compartment.

Duration	Water	0.63 μM	6.3 μM	63 μM	630 μM
*n*	35	30	75	20	20
Mean	1,114	1853	2,161	1752	901.1
SD	2004	1738	2,282	1810	2,154
SEM	360	293.8	272.7	404.7	481.6
Min	0	0	0	0	0
Max	7,177	7,178	7,177	6,641	6,813
Range	7,177	7,178	7,177	6,641	6,813

**Table 2B:** Statistical analysis of nicotine dose-dependency - frequency in nicotine compartment.

Duration	Water	0.63 μM	6.3 μM	63 μM	630 μM
*n*	35	30	75	20	20
Mean	6	13	6	7	2
SD	7	12	8	9	2
SEM	1	2	0.9	2	0.5
Min	0	0	0	0	0
Max	23	44	46	34	9
Range	23	44	46	34	9

Before adding nicotine to the nicotine compartment or water in case of the control, no statistically significant differences were detected for the cumulative duration (Figures 3A,C) in different test groups in the nicotine compartment (water, *n* = 35; 0.63 μM nicotine, *n* = 30; 6.3 μM nicotine, *n* = 75; 63 μM nicotine, *n* = 20; and 630 μM nicotine, *n* = 20). Cumulative durations in the nicotine compartment during the 30-min phase prior to adding nicotine were not significantly different between test groups (Figure 3A). Compared to the water control group (mean 355.6 s; SEM ± 96.94; *n* = 35), the mean cumulative durations at 0.63 μM nicotine (563.4 s; SEM ± 126.5 s; *n* = 30), at 6.3 μM nicotine (405.6 s; SEM ± 73.94; *n* = 75), at 63 μM nicotine (629.6 s; SEM ± 161.6; *n* = 20) and at 630 μM nicotine (mean 527.2 s; SEM ± 148.4; *n* = 20) were not significantly different (*p* > 0.9999 for water vs. 0.63 μM, water vs. 6.3 μM, and water vs. 63 μM; *p* = 0.0909 for water vs. 630 μM; one-way ANOVA; Kruskal–Wallis with Dunn’s multiple comparison test).

Cumulative durations in the nicotine compartment significantly increased after addition of nicotine in the 0.63 and 6.3 μM nicotine groups (Figure 3B). Compared to the water control (mean 1,056 s, SEM ± 323.2, *n* = 35), 0.63 and 6.3 μM nicotine increased the cumulative time in the nicotine compartment significantly (0.63 μM: mean 1853 s; SEM ± 293.8; *n* = 35; 6.3 μM – mean 2,222 s, SEM ± 277.3; *n* = 75). At 63 μM nicotine the cumulative time (1752 s ± SEM 404.7; *n* = 20) was longer compared to the mean of the water control experiment but was not significantly different potentially because of the wide range (Figure 3B and Table 2A). At 630 μM nicotine the mean cumulative time in the nicotine compartment was reduced (901.1 s; SEM ± 481.6; *n* = 20) compared to the water controls, but not significantly different from the water control group (Figure 3B). Overall, nicotine concentrations ranging from 0.63 to 630 μM significantly increased the cumulative time spent in the nicotine compartment, while higher concentrations did not affect the time spent in the nicotine compartment. The differences between water controls and 0.63 μM nicotine (*p* = 0.0158) and between controls and 6.3 μM nicotine (*p* = 0.0092) were significant. Calculated means for 63 and 630 μM nicotine were not significantly different from the mean of the water control. Cumulative durations for the different test groups in the water compartments before (Figure 3C) and after nicotine addition (Figure 3D) were not significantly different from the water controls at all tested nicotine concentrations (0.63, 6.3, 63, and 630 μM).

The different nicotine concentrations resulted in fewer changes in the number of entrances into the nicotine compartment compared to the duration (Figures 3F,H and Table 2B). Before the addition of nicotine into the nicotine compartment and during 30 min pre-nicotine phase entrances into the water compartment (mean 2; SEM ± 1; *n* = 35), the nicotine compartment at 0.63 μM (mean 3; SEM ± 1; *n* = 30), the nicotine compartment at 6.3 μM (mean 2; SEM ± 0; *n* = 75), the nicotine compartment at 63 μM (mean 2; SEM ± 1; *n* = 20) and the nicotine compartment at 630 μM (mean 2; SEM ± 0; *n* = 20) were not significantly different (Figure 3E). After the addition of nicotine (Figure 3F) and over the 2-h nicotine phase, significantly more entrances occurred at 0.63 μM nicotine (mean 13; SEM ± 2; *n* = 30; *p* = 0.0478) and at 6.3 μM nicotine (mean 6; SEM ± 0.9; *n* = 75), but not at 63 μM nicotine (mean 7; SEM ± 2; *n* = 20) and 630 μM nicotine (mean 2; SEM ± 0.5; *n* = 20) compared to the number of entrances in water controls (mean 6; SEM ± 1; *n* = 35). The number of entrances into the water compartment in controls before addition of nicotine (mean 6; SEM ± 0.9; *n* = 35), at 0.63 μM nicotine (mean 10; SEM ± 2; *n* = 30), 6.3 μM nicotine (mean 7; SEM ± 1; *n* = 75), 63 μM nicotine (mean 10; SEM ± 4; *n* = 20) and 630 μM nicotine (mean 4; SEM ± 0.8; *n* = 20) did not differ significantly (*p* > 0.9999) (Figure 3G). Addition of nicotine did not result in significant differences (*p* > 0.9999) in the frequency of entering the water compartment between water (mean, SEM ± 0.9, *n* = 35), 0.63 μM nicotine (mean 10, SEM ± 2, *n* = 30), 6.3 μM nicotine (mean 7, SEM ± 1, *n* = 75), 63 μM nicotine (mean 10, SEM ± 4, *n* = 20) and 630 μM nicotine (mean 4, SEM ± 0.8, *n* = 20) (Figure 3H). Overall, the frequency of entering nicotine and water compartments did not change significantly with nicotine concentrations, except for the nicotine compartment at 0.63 and 6.3 μM nicotine.

The analysis of the percentages of behavioral phenotypes (Figure 3I) showed that nicotine caused the highest percentages of seekers for 0.63 μM nicotine (30%) and 6.3 μM nicotine (26.7%). Fewer nicotine-seekers were identified in water controls (20%) and at nicotine concentrations of 63 μM (20.0%) and 630 μM nicotine (20.0%). The percentage of nicotine avoiders was the lowest at 0.63 μM nicotine (16.7%) and highest at 63 μM nicotine (35.0%). In control experiments in which water was added to the test compartment, 22.9% of tested larval zebrafish were avoiders. Percentages of non-seekers were 57.1% for water controls, 53.3% for 0.63 μM nicotine, 45.3% for 6.3 μM nicotine, 45.0% for 63 μM nicotine and 50% for 630 μM nicotine. Thus, lower nicotine concentrations (0.63–6.3 μM) caused more nicotine-seeking while higher concentrations (63, 630 μM) were associated with more nicotine avoidance.

### 3.4. Repeated testing

**Figure 4:**
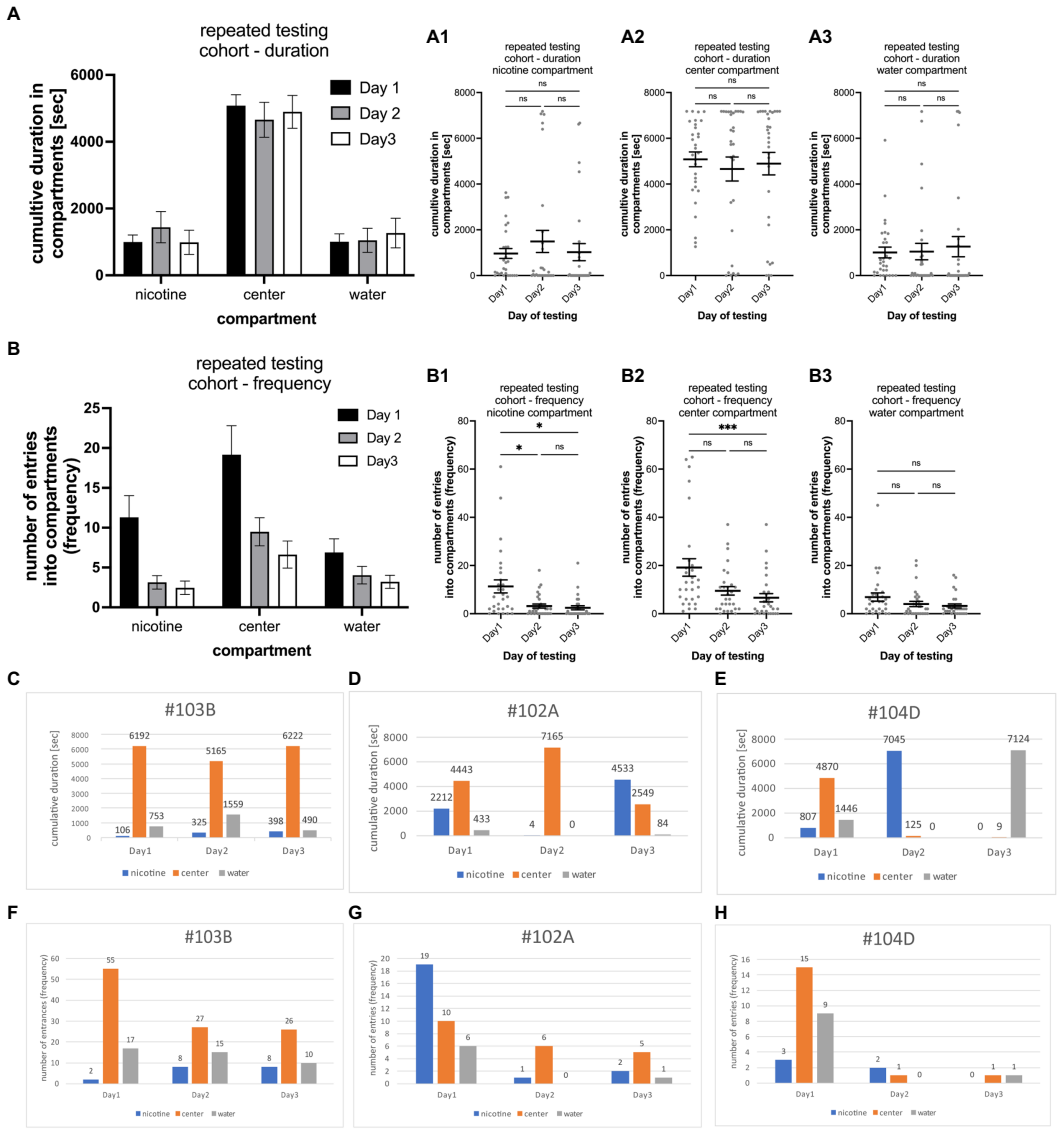
Shows the results of repeated testing of individual larval zebrafish for nicotine-seeking and avoidance in the gradient maze over the course of 3 days. **(A)** Cumulative durations in the nicotine, center and water compartments of the entire cohort (*n* = 29) show now similar behavioral activities on all 3 days [two way ANOVA; *F*(2,261) = 0.002750; *p* = 0.9973; *n* = 29] **(A1–A3)** show that the cumulative times for each compartment [**(A1)** nicotine compartment; **(A2)** center compartment; **(A3)** water compartment] were similar with no significant (ns) differences. **(B)** The number of entrances into the compartments was higher on the first day of testing for all three compartments (nicotine, center, and water). On day 2 and 3 of the testing lower number of entrances were recorded than on day 1 [two way ANOVA; *F*(2,252) = 16.44; *p* < 0.0001; *n* = 29]. **(B1)** The number of entrances into the nicotine compartment was significantly higher on day 1 in comparison to day 2 and 3 (day1 vs. day2, *p* = 0.0198; day1 vs. day 3, *p* = 0.0145). **(B2)** Larval zebrafish also entered the center compartment more frequently on day 1 compared to day 3 of testing (*p* = 0.0005). **(B3)** The number of entrances into the water compartment were not different on all three testing days. Individual data points are shown with the mean ±SEM. Statistical significance was tested using a one-way ANOVA Kruskal–Wallis test with Dunn’s comparison test. Representative examples of larval zebrafish that showed similar behavioral profiles on all three testing days [#103B, **(C)** duration, **(F)** frequency], on two of the three testing days [#102A, **(D)** duration, **(G)** frequency]. In the third example (#104D, **E,H**) different behavioral profiles were recorded on each day of testing. Numbers on top of bars represent the cumulative duration spent in the compartment in (**C–E)** and the number of entrances into the compartment in **(F–H)**. ***p < 0.0005; *p < 0.0198.

To determine the consistency of nicotine seeking and avoidance behavior, 30 individual larval zebrafish were tested repeatedly over 3 days at 6, 7, and 8 dpf, one time on each day at the same time of the day and at a concentration of 0.63 μM nicotine. Results from one larval zebrafish on 1 day were not included because of a fly that entered the maze. The analysis of the cumulative durations of the entire cohort on each tested day did not show significant differences [two way ANOVA; *F*(2,261) = 0.002750; *p* = 0.9973; *n* = 29] (Figure 4A). Larval zebrafish spent similar times in the nicotine compartment on day 1 (Figure 4A1) (963.5 s; SEM ± 215.8; *n* = 29), day 2 (1,491 s; SEM ± 482.0; *n* = 29), and day 3 (1,021 s; SEM ± 373.0; *n* = 29). These durations were not significantly different between day 1 and day 2 (*p* = 0.7706, n = 29), day1 and day3 (*p* = 0.935, *n* = 29) and day2 and day 3 (*p* = 0.9214; n = 29). Cumulative durations for the center compartment (Figure 4A2) were higher compared to the nicotine compartment on day 1 (5,080 s; SEM ± 325.0; *n* = 29) day 2 (4,658 s; SEM ± 523.5; *n* = 29), and day 3 (4,894 s; SEM ± 491.1; *n* = 29). No differences were seen for the cumulative durations between day1 and day 2 (*p* ≥ 0.9999, *n* = 29), day 1 and day 3 (*p* > 0.9999, *n* = 29) and day 2 and day 3 (*p* > 0.9999, *n* = 29). The cumulative durations for the water compartment also were not significantly different (Figure 4A3) on day1 (1,007 s; SEM ± 235.7; *n* = 29), day 2 (1,047 s; SEM ± 359.7; *n* = 29) and day 3 (1,265 s; SEM ± 441.7; *n* = 29). Comparisons of cumulative durations in the water compartment between day 1 and day 2 (0.3852, *n* = 29), day 1 and day 3 (*p* = 0.2258, *n* = 29) and day 2 and day 3 (*p* > 0.9999, *n* = 29) did not indicate significant differences. The analysis of the frequency of entering compartments for the entire cohort on each tested day showed a significant shift in activity [two way ANOVA; *F*(2,252) = 16.44; *p* < 0.0001; *n* = 29] (Figure 4B). Larval zebrafish entered compartments more frequently on the first day and less on the second and third day of testing (Figures 4B1–B3). The frequency of entering the nicotine compartment (Figure 4B1) was the highest on day1 (11.3, SEM ± 2.7; *n* = 29) compared to day2 (3.1; SEM ± 0.8, *n* = 29) and day 3 (2.5; SEM ± 0.8, *n* = 29). The frequency on day 1 was significantly higher compared to day 2 (*p* = 0.0198, *n* = 29) and day 3 (*p* = 0.145, *n* = 29). No significant change was found between day 2 and day 3 (*p* = 0.8527, *n* = 29). The difference between entrances into the center compartment (Figure 4B2) also were the highest on the first test day (19.2; SEM ± 3.6; *n* = 29) compared to the second (9.4; SEM ± 1.8, *n* = 29) and third day (9.1; SEM ± 1.7 n = 29). The differences between day 1 and day 3 were statistically significant (*p* = 0.0005) but not between day 1 and day 2 (*p* = 0.1026) and between day 2 and 3 day 3 (*p* = 0.2975). Frequency of entrances into the water compartment (Figure 4B3) were not significantly different on all days (Day1 vs. Day2 *p* = 0.1762; day1 vs. day 2 *p* = 0.1773; day 2 vs. day 3 *p* > 0.9999). The results of repeated experiments showed a high degree of consistency for the cumulative duration on all three test days. The frequency of entering compartments were similar on day 2 and day 3 of testing with less movement between compartments compared to test in day 1 when movement activity between compartments was about twice as high as on day 2 and 3. Otherwise, major shifts between days to or from the nicotine and water compartments were not detected for the cohort.

Variations of the behavioral phenotype – seeker vs. avoider – were found in individual zebrafish. Overall, the behavioral phenotype was consistent on two or more days in 79.3% of tested larval zebrafish. Of the 29 tested larval zebrafish, 11 (37.9%) showed the same seeking behavior on all 3 days (example #103B in Figures 4C,F), 12 (41.4%) showed the same behavior on 2 out of 3 days (example #102A in Figures 4D,G), and 6 (20.7%) showed different behavioral phenotypes on all 3 days (example #104D in Figures 4E,H). The larval zebrafish that had a similar behavioral phenotype based on duration on 2 out of 3 days included 5 that were non-seekers on 2 days and seekers on 1 day, 2 larval zebrafish that were seekers on 2 days and non-seekers on 1 day, 2 larval zebrafish that were avoiders on 1 day and non-seekers on 2 days and 2 larval zebrafish that were avoiders on 2 days and non-seekers on 1 day. The behavioral phenotypes for frequency were like those for duration in examples #103B and #104D. Example #102A was a frequency seeker on day1 of testing, a duration seeker on day 3 of testing, and a non-seeker on day2 of testing. Testing larval zebrafish throughout their larval life (up to 30 dpf) was challenging because of high mortality rates. Individual larval zebrafish showed more consistency than variation in their behavioral phenotypes in repeated three-choice gradient maze tests which contributed to the overall consistency in the cohort results. However, individual differences and switching of nicotine preferences have been observed and could be based on individual genomic or developmental variations.

### 3.5. Treatment with nicotine and varenicline changes nicotine-seeking and avoidance behavior

#### 3.5.1. Nicotine pre-treatment

**Figure 5:**
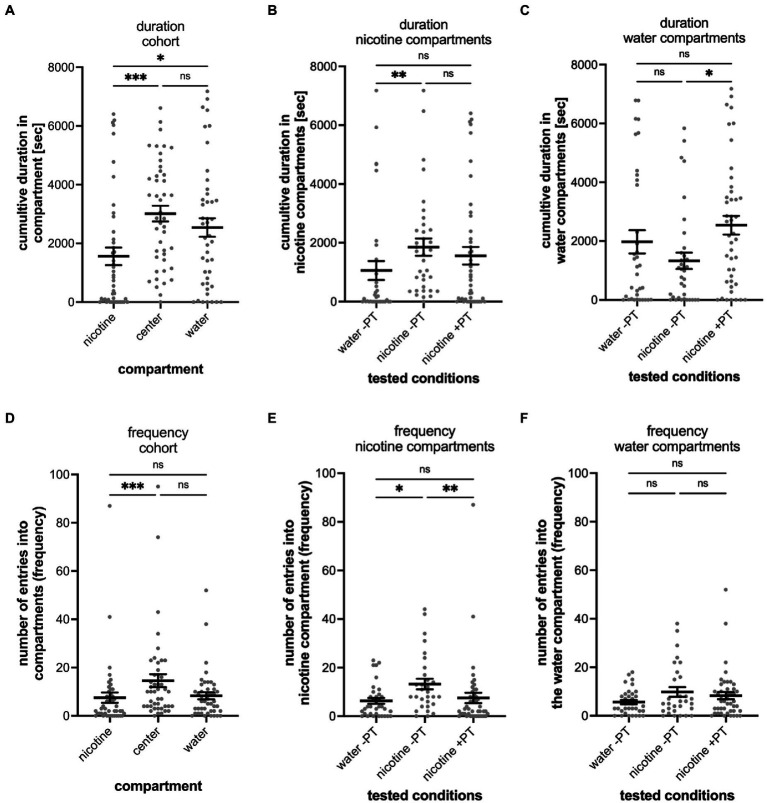
Shows the results of nicotine pre-treatment (PT) experiments. **(A)** The results of the entire tested cohort (*n* = 45) are shown for the cumulative duration spent in compartments and the number of entrances into compartment (frequency; D). **(B)** Compares the durations spent in the nicotine compartment under different experimental conditions including water controls without nicotine pretreatment when water was added to the “nicotine compartment” (water -PT, *n* = 35), at a 0.63 μM nicotine concentration in the nicotine compartment without nicotine pretreatment (nicotine -PT, *n* = 30), and at 0.63 μM nicotine in the nicotine compartment with a 1 μM nicotine pretreatment (nicotine +PT, *n* = 45). The cumulative duration increased in nicotine without pre-treatment compared to water (*p* = 0.0014) but did not change with nicotine-pretreatment (*p* = 0.1124). **(C)** Comparison of the cumulative time spent in the water compartment under the indicated experimental conditions as in **(B)** (water –PT, nicotine –PT, and nicotine +PT). Nicotine pre-treatment increased the duration spent in the water compartment (*p* = 0.0287). **(D)** The results of the entire tested cohort (*n* = 45) are shown for the number of entrances into the nicotine, center, and water compartments (frequency). The number of entrances into the nicotine compartment (nicotine) were lower compared to the entrances into the center compartment (*p* = 0.0008). Entrances into the water compartment compared to the center compartment were not significantly different (*p* = 0.0851). **(E)** Comparison of the number of entrances (frequency) into the nicotine compartment under the same indicated condition as in **(B)** (water –PT, nicotine –PT, and nicotine +PT). The number of entrances increased (*p* = 0.0175) when nicotine was added into the nicotine compartment without nicotine pre-treatment (nicotine –PT) compared to water controls (water –PT) but not with a nicotine pretreatment (nicotine +PT) (*p* = 0.3816). Nicotine pretreatment reduced the number of entrances into the nicotine compartment (nicotine +PT) when compared to the nicotine without nicotine pretreatment (nicotine -PT) (*p* = 0.0036). **(F)** Entrances into the water compartment under the three different experimental conditions (water –PT, nicotine –PT, and nicotine +PT) were not significantly different. Individual data points are shown with means ±SEM. Statistical significance was tested using a Kruskal–Wallis test with Dunn’s comparison test. ***p < 0.0008; **p < 0.0036, *p < 0.0364; ns > 0.0851.

Since exposure to nicotine in preferred place preference tests of adult zebrafish results in behavioral changes, we carried out nicotine-pretreatment experiments in the gradient maze to determine if nicotine-seeking and avoidance behavior could change with nicotine exposure. Overall, the pre-treatment seemed to affect the seeking and avoidance behavior only weakly. The mean time of the entire cohort spent in the water compartment (2,538 s; SEM ± 316, *n* = 45), compared to the nicotine compartment (mean 1,558 s; SEM ± 301; *n* = 45) was significantly longer (*p* = 0.0364) and similar to the cumulative time spent in the center compartment (Figure 5A). The cumulative times spent in the nicotine compartment (Figure 5B) between not-pretreated (–PT) and pretreated (+PT) larval zebrafish was similar (not pretreated – 1853 s, SEM ± 293.8; *n* = 35; pretreated- 1,558 s; SEM ± 300.6; n = 45) and not significantly different (*p* = 0.1124). Compared to the water control group (mean 1,056 s; SEM ± 323.3, *n* = 35), nicotine alone without pretreatment resulted in significantly more cumulative time spent in the nicotine compartment (*p* = 0.0014) but nicotine pretreatment did not (*p* = 0.3143). The cumulative time spent in the water compartment by the not pretreated nicotine group (nic -PT, mean 1,329 s, SEM ± 278.7, *n* = 35) was significantly lower compared to the nicotine pre-treatment group (2,538 s, SEM ± 316.0, *n* = 45) (*p* = 0.0287) (Figure 5C). However, the mean cumulative duration in the water compartment of pre-treated larval zebrafish (nicotine + PT) was not significantly different from water controls (water –PT; mean 1975 s, SEM ± 395.9, *n* = 35) (*p* = 0.3816). Thus, the nicotine pre-treatment did not change the time spent in nicotine or water compartments clearly.

Result of entrances into the gradient maze compartments showed similar patterns as results for the cumulative duration and provided no strong indication of a shift in nicotine-seeking or avoidance behavior. The entire cohort of larval zebrafish in the nicotine pre-treatment experiments entered the center compartment most frequently (mean number of entrances 15; SEM ± 3) and both the nicotine compartment (mean 8; SEM ± 2) and the water compartment (mean 8; SEM ± 1) at similar frequencies (Figure 5D). The center compartment was entered more frequently than the nicotine compartment (*p* = 0.008). But there was no significant difference between entrances into the center and water compartments. Nicotine pretreatment reduced the number of entrances into the nicotine compartment significantly (not pretreated vs. pretreated *p* = 0.0175, Figure 5E) as the mean number of entrances into the nicotine compartment by treated larval zebrafish (mean 7.5; SEM ± 2.1; *n* = 45) was lower compared to not pretreated larval zebrafish (mean 13.23; SEM ± 2.1; *n* = 30) and levels more similar to water controls without pretreatment (mean 6.314; SEM ± 1.2; *n* = 35). Number of entrances into the water compartment in water controls (mean 5.7, SEM ± 0.9, *n* = 35), nicotine not pretreated (mean 9.833, SEM ± 1.974, *n* = 30) and nicotine pretreated groups (mean 8.311, SEM ± 1.5, *n* = 45) did not differ significantly (Figure 5F).

The nicotine pretreatment did not shift the percentage of nicotine-seekers compared to other test groups (Figure 3I). Out of 45 tested larval zebrafish 9 (20%) were identified as nicotine seekers (4 super seekers, 4 duration seekers, 1 frequency seeker). In contrast, the percentage of nicotine avoiders was higher 35.6% (16 out of 45 tested larval zebrafish). The 16 nicotine-avoiding larval zebrafish included 9 duration avoiders, 3 frequency avoiders and 4 super avoiders.

Overall, the nicotine pre-treatment facilitated only slight changes more toward nicotine avoidance behavior and a larger percentage of nicotine avoiders but that shift was not significant when compared to not nicotine pretreated larval zebrafish.

#### 3.5.2. Varenicline treatment

**Figure 6:**
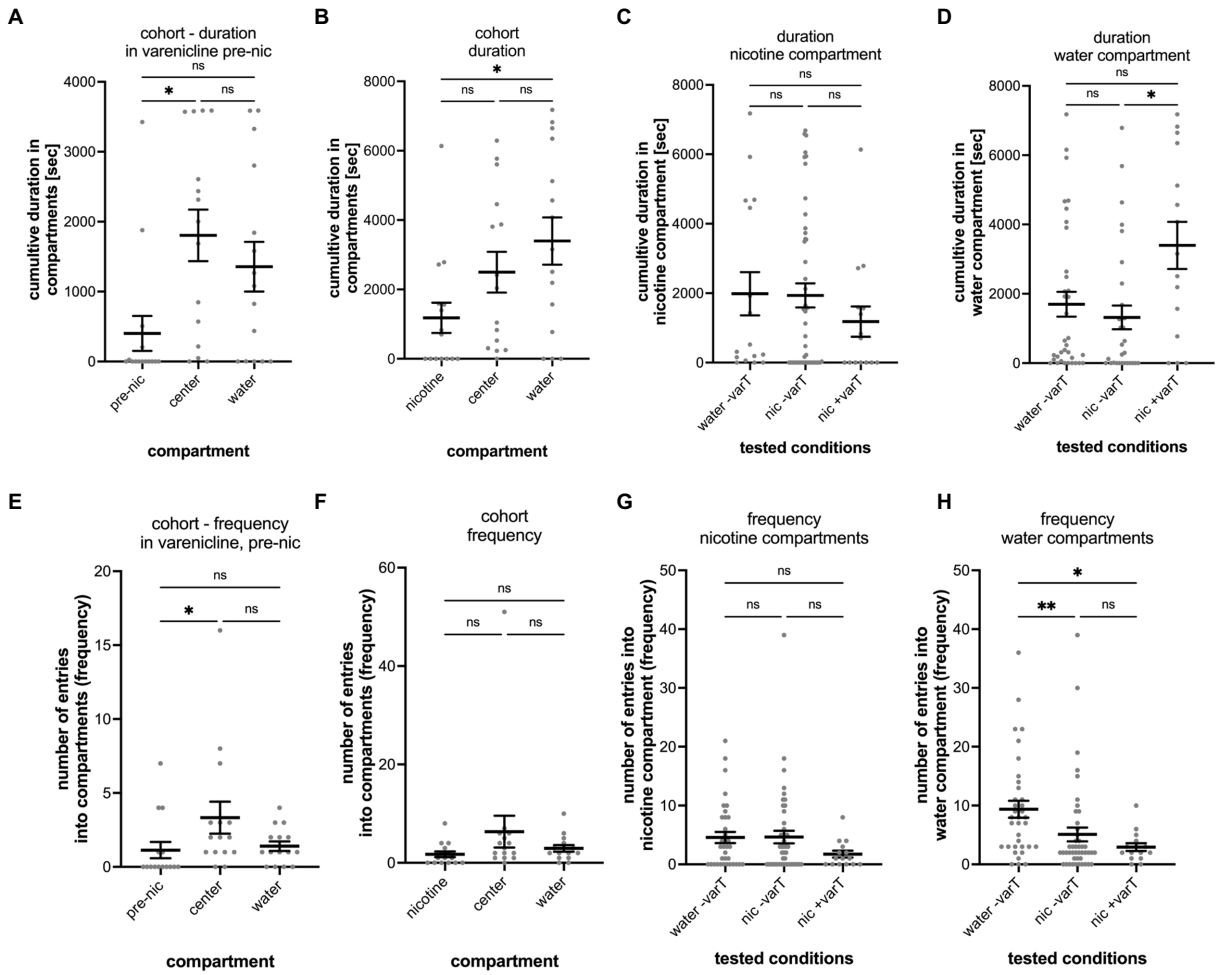
Analysis of varenicline treatment experiments. The results of the entire cohort (*n* = 15) are shown in **(A)** for the cumulative duration in the nicotine (pre-nic), center and water compartments during the 1-h varenicline phase before addition of nicotine into the nicotine compartment (pre-nic). The difference between the cumulative duration in the nicotine and center compartment was significant (*p* = 0.0037) but not between the center compartment and the water compartment (*p* = 0.9581) and the pre-nicotine compartment and the water compartment (*p* = 0.0766). **(B)** Shows the cumulative duration in the nicotine nicotine, center, and water compartments in varenicline after addition of nicotine into the nicotine compartment (nicotine). The cumulative duration in the water increased (nicotine vs. water, *p* = 0.0307) whereas the duration in the center compartment decreased (nicotine vs. center, *p* = 0.2432) and was not significantly different as before the addition of nicotine. **(C)** No difference was found between the three experimental conditions for the cumulative duration in the nicotine compartment. **(D)** The cumulative duration in the water compartment in varenicline and after nicotine addition (nic + varT) was significantly larger than the without varenicline (nic -varT) (*p* = 0.0198). **(E)** Comparison of the number of entrances (frequency) into the nicotine, center and water compartment during varenicline treatment and before addition of nicotine shows a significant difference between the nicotine and center compartment (*p* = 0.0242). **(F)** Addition of nicotine eliminated this difference. **(G)** No differences for the number of entrances into the nicotine compartment were found when comparing the different treatments. **(H)** Nicotine decreased the number of entrances into the water compartment without (water –varT vs. nic –varT, *p* = 0.0021) and with varenicline treatment (nic –varT vs. nic + varT; *p* = 0.0161). Varenicline did not change the number of entrances with nicotine in the nicotine compartment (*p* = 0.6111). Individual data points are shown with the mean ±SEM. Statistical significance was tested using a Kruskal–Wallis test with Dunn’s comparison test. **p < 0.0037; *p < 0.0307; ns > 0.0766.

Varenicline is the active substance in the smoking cessation drug Chantix that reduces nicotine craving (Coe et al., 2005). To explore whether varenicline could change nicotine-seeking and avoidance behavior in the gradient maze test, larval zebrafish were treated with varenicline (20 μM) for 1 h in the gradient maze before the nicotine was added to the maze compartments. Moreover, varenicline has been used at concentrations up to 50 μM for larval zebrafish in overnight treatment (Cousin et al., 2014). We used 20 μM varenicline, because this concentration resulted in significant reduction of movement activity in acute nicotine response tests without indication of detrimental effects (Schneider, unpublished results). Over the first hour of the experiment, varenicline was added directly to the gradient maze and movement activity of larval zebrafish was recorded for 1 h. Nicotine was applied to the nicotine compartment to reach a final concentration around 3.15 μM (based on calculations of concentrations described above for 5 μl of 1 mM nicotine added) and in between the effective nicotine concentrations of 0.63 and 6.3 μM as shown in the dose-dependency experiments (Figure 3B). During the varenicline treatment phase, larval zebrafish spent most of the time in the center compartment (1804 s; SEM ± 368.7; *n* = 15) and significantly less time (*p* = 0.0037, *n* = 15) in the “nicotine compartment” (402.7 s; SEM ± 250.0; *n* = 15) (Figure 6A). The cumulative duration between times spent in the center and the water compartment (1,356 s; SEM ± 356.1; *n* = 15) were not significantly different (*p* = 0.9581, *n* = 15). After addition of nicotine to a final concentration of 3.15 μM and the 2-h recording in the presence of both nicotine and varenicline, the cumulative duration spent in the water compartment increased (3,397 s, SEM ± 680.6, *n* = 15) while the cumulative time spent in the center compartment (2,496 s, SEM ± 587.3, *n* = 15) decreased for the cohort (Figure 6B) making the difference in cumulative duration between the water compartment and the nicotine compartment (1,182 s SEM ± 436.3, *n* = 15) significant (Nicotine vs. center: *p* = 0.2432, *n* = 15; nicotine vs. water: *p* = 0.0307, *n* = 15; center vs. water *p* > 0.9999, *n* = 15). The cumulative duration after adding nicotine in the nicotine compartment (1,182 s; SEM ± 436 s), water controls (1983; SEM ± 624 s) and untreated larval zebrafish (1935; SEM ± 348 s) did not indicate significant differences (Figure 6C). In addition, the cumulative time in the water compartment of varenicline treated larval zebrafish (nic + varT: 3397 s; SEM ± 681) had significantly increased (*p* = 0.0198) compared to untreated larval zebrafish (nic -varT: 1729 s; SEM ± 414) (Figure 6D).

During the varenicline treatment and before addition of nicotine the number of entrances into the nicotine compartment were significantly lower (1.13; SEM ± 0.551; *n* = 15) compared to the center compartment (3.33; SEM ± 10.8; *n* = 15) (*p* = 0.0242, *n* = 15) (Figure 6E). The addition of nicotine to the nicotine compartment resulted in number of entrances (nicotine: 1.733 SEM ± 0.5812, *n* = 15, center: 6.333 SEM ± 3.238, *n* = 15; water: 2.933 SEM ± 0.6652, *n* = 15) that were not significantly different from each other (nicotine vs. center: *p* = 0.0771, *n* = 15; nicotine vs. water: *p* = 0.3352, *n* = 15; center vs. water: *p* > 0.9999, *n* = 15).

The number of entrances into the three compartments of the varenicline treated group (*n* = 15) were not significantly different and varied between 1.7 (SEM ± 0.5812) in the nicotine compartment, 6.3 in the center compartment (SEM ± 3.238) and 2.9 (SEM ± 0.6652) in the water compartment (Figure 6F). The number of entrances into nicotine compartments between varenicline treated and un-treated groups were also not significantly different (Figure 6G). Compared to the number of entrances into the water compartment in controls (water added to the nicotine compartment, no varenicline treatment) the number of entrances into water compartments was significantly lower after nicotine had been added in both varenicline untreated (−varT, *p* = 0.0021; *n* = 15) and varenicline treated (+varT, *p* = 0.0161, *n* = 15) tests (Figure 6H). Varenicline treatment did not change the number of entrances into the water compartment after nicotine had been added (*p* > 0.9999, *n* = 15).

The varenicline treatment before the addition of nicotine, generated 20% seekers, 40% avoiders and 40% non-seekers. The addition of nicotine to the nicotine compartment in the presence of varenicline resulted in an increase in the percentage of avoiders from 40 to 60%, a decrease of the percentage of nicotine seekers from 20 to 13.3% and a decrease of non-seekers from 40 to 26.7%.

## 4. Discussion

### 4.1. Testing individual larval zebrafish

The study presents a new three-choice behavioral assay for measuring responses of larval zebrafish to chemicals such as nicotine. The new approach of measuring the behavioral responses of individual larval zebrafish in a three-choice assay generated robust and reproducible results and demonstrated that individuals within one cohort differ in their response to nicotine by seeking, avoiding and non-seeking. The mazes were kept small so that larval zebrafish could easily explore the entire maze during recording periods. By separating three compartments by narrow links, we could contain the diffusion of nicotine and generate one nicotine compartment on one side and a compartment without nicotine on the other. The gradient design has been applied successfully in a copper avoidance test for zebrafish (Araújo et al., 2019). Based on food dye diffusion experiments some nicotine it likely to diffuse into the center compartment but to a much lesser degree into the water compartment connected to the center compartment. The measurement of absorbance of nicotine at 260 nm also supports that nicotine is contained in the nicotine compartment over the 2-h nicotine phase of the experiments. Because of the low sensitivity of the spectrophotometric assay, some diffusion into the center compartment or the water compartment cannot be ruled out, but the highest concentration of nicotine would be in the nicotine compartment and the lowest in the water compartment. The link between compartments was wide enough for larval zebrafish to turn around in the link. Thus, freely moving larval zebrafish had the choice to enter the water and nicotine-free compartment. The observational period was kept at 2 h to allow neuronal cell signaling mechanisms and potential gene expression changes to contribute to the nicotine seeking behavior (Kily et al., 2008). Tracking the path of larval zebrafish in the maze and calculating the time spent in the nicotine compartment allowed the differentiation of behavioral phenotypes of nicotine seeker, avoider and non-seeker in addition to a quantitative analysis of the test groups. Previously, choice experiments were used to measure the preference for morphine (Bretaud et al., 2007) and nicotine (Krishnan et al., 2014) in larval zebrafish. In both studies larval zebrafish could swim freely within the test chamber. However, groups of larval zebrafish were used for the morphine study. Once introduced into a continuous rectangular choice chamber (25 cm × 16.5 cm × 6.5 cm; L × W × H), morphine was delivered on one end and water on the opposite end. A drain in the center of the chamber reduced the diffusion of morphine in the water compartment. Snapshots were taken in 10 s intervals and the number of larval zebrafish in each compartment was used for the analysis. Using this approach, it remains unclear if the same larval zebrafish spent more time in the morphine compartment or if different larval zebrafish explored the morphine compartment for a short period. Individual variations were not accounted for. In a different experiment (Krishnan et al., 2014) nicotine was delivered and contained *via* microfluidic pump system in a corner of a rectangular test chamber (76 mm × 32 mm × 30 mm (L × W × H). While no physical barriers interfered with the movement of the single larval zebrafish in the experiment, the test chamber was virtually divided into the areas for the analysis of experiments that used distance between the larval zebrafish and the source of fluid delivery as a criterion for attraction and avoidance of the chemical. The three-choice behavioral test chamber introduced in our study is suitable for measuring behavioral responses to nicotine of individual freely swimming larval zebrafish and has the advantages of scalability and simple delivery of nicotine in a restricted area of the maze without the need for special perfusion systems. Both nicotine-seeking and avoiding larval zebrafish could be identified and significant responses to changes in nicotine concentrations could be observed in individual larval zebrafish. By measuring the behavior of single larval zebrafish, in contrast to groups (Bretaud et al., 2007) individual variations in behavioral responses could be measured in future studies.

### 4.2. Nicotine-seeking and avoidance behavior

The results show that a subset of ~20–30% of larval zebrafish spent most of the time the nicotine-containing compartment, a behavior that we named nicotine-seeking. That 20–30% of larval zebrafish preferred the nicotine compartment under our experimental conditions appears to be dependent on the concentration of nicotine. Along the same lines, nicotine avoidance behavior is also apparent in a subset of larval zebrafish within a cohort. Such mixed variations in individual behavioral responses are common. Self-administration experiments of nicotine (and other drugs of dependence) using mice demonstrated differences among strains of mice. In zebrafish, differences of inhibitory avoidance behavior, cortisol levels and gene expression between the AB and TL strains of zebrafish have been described (Gorissen et al., 2015). Locomotor activity has been described to vary consistently between individual adult zebrafish (Tran and Gerlai, 2013). Swimming activity of adult zebrafish measured by distance traveled varied between low, medium and high activity fish. In addition, females travelled longer distances than males (Tran and Gerlai, 2013). Moreover, individual variations have been described in zebrafish for the exposure to alcohol (Araujo-Silva et al., 2018), attraction and avoidance of odorants (Krishnan et al., 2014), exploratory behavior (Rajput et al., 2022), and learning and memory (Gerlai, 2016). Moreover, repeated testing in the gradient maze showed a certain degree of consistency indicating that nicotine-seeking and avoidance behavior is not random but switching of preferences has been observed. Thus, the characterization of nicotine-seekers, avoiders and non-seekers or variation in nicotine seeking and avoidance behavior among individual larval zebrafish described here are in line with individual variations reported in several experimental approaches. Since nicotine preference and avoidance are known to be associated with different neuronal circuits (Fowler and Kenny, 2014), the three-choice assay might be used to study the neuronal dynamics between seeking and avoidance. The application of nicotine to the nicotine compartment could be improved in future experiments.

### 4.3. Larval zebrafish respond to different nicotine concentrations

The behavioral choice experiment allows larval zebrafish to choose or avoid nicotine and adjust their nicotine uptake in a self-administration-like mode. The nicotine concentration in the compartment plays a critical role for the separation of seeking and avoidance behavior. The shifting of seeking and avoidance behavior with concentrations in the nicotine compartment is an indication that the exposure to nicotine is controlled by an underlying mechanism and does not occur randomly. Similar to studies in rodents, the behavioral response of larval zebrafish in the three-choice test follows an inverted-U shape with stronger nicotine seeking occurring at lower nicotine concentrations (0.63, 6.3 μM) and nicotine avoidance at higher nicotine concentrations (63 μM, 630 μM). A similar relationship has been described for larval zebrafish in acute nicotine response tests (Petzold et al., 2009). These results could indicate that larval zebrafish titrate their nicotine intake and actively adjust their exposure to nicotine by moving between compartments. The cumulative time spent in the nicotine compartment and the percentage of nicotine seekers correlates stronger with an inverted U-shaped dose–response curve, but not the cumulative time and the number of entrances into the water compartment. In rodents, the inverted U-shaped relationships between nicotine concentration and behavioral responses is robust (e.g., Fowler and Kenny, 2014) and has been also described for conditioned place preference tests, for example (Braida et al., 2020). Aversion behavior also follows an inverted-U shaped dose–response curve over higher nicotine concentrations. Nicotine avoidance has been described in mice (Fowler and Kenny, 2014) and has been suggested in larval zebrafish (Krishnan et al., 2014). While a clear correlation between the nicotine-concentration and the percentage of nicotine seekers has been found in form of an inverted-U shaped curve, the number of entrances into the nicotine compartment is only weakly correlated with the nicotine concentration. A correlation between the cumulative time spent in the water compartment is absent. The relationship and dynamic between time spent in a compartment vs. entering a compartment could indicate separate regulatory entities or neural circuits. Overall, the nicotine dose–response relationships measured in the nicotine-seeking and avoidance assay align with the typical nicotine dose-relationships reported for rodents and zebrafish.

### 4.4. Varenicline-induced changes in nicotine-seeking and avoidance

Varenicline represents the gold standard of nicotine cessation treatment as it is widely used in smoking cessation treatment and reduces craving for smoking cigarettes (Ebbert et al., 2010). That larval zebrafish showed stronger nicotine avoidance behavior when treated with varenicline supports the use of the three-choice gradient maze for screening of potential pharmacotherapeutics for improved nicotine cessation treatment. An increase in nicotine avoidance aligns with a reduced nicotine exposure and intake. Alternatively, increased avoidance could be associated with increased desensitization of acetylcholine receptors at high concentrations of varenicline (Ortiz et al., 2012). The zebrafish genome contains nicotinic alpha4 and beta2 nicotinic acetylcholine receptor genes that interact with varenicline (Klee et al., 2012). Both genes of the alpha4 and beta2 nicotinic acetylcholine receptor subunit are expressed in the brain of larval zebrafish (Ackerman et al., 2009; Garcia-Gonzalez et al., 2020). In conditioned place preference tests using adult zebrafish, varenicline reduced the time spent in the nicotine-paired side of the behavioral chamber (Ponzoni et al., 2014). Rodents have been used in pre-clinical experiments for the study of varenicline actions. Self-administration of nicotine was reduced by varenicline treatment in rats (Scuppa et al., 2015; Zheng-Ming et al., 2021). Varenicline has been shown in rodents to decrease nicotine-induced hyperlocomotion, reduce nicotine-induced sensitization and improve the performance times in the Morris water maze (Zaniewska et al., 2008; King et al., 2011). Thus, the increased nicotine avoidance behavior in varenicline treated larval zebrafish in the gradient maze aligns with results obtained in adult zebrafish and rodent models.

That the response to nicotine at an early developmental stage of zebrafish (6–8 dpf) could be dependent on previous nicotine-encounters is only weakly supported by results from nicotine-pretreatment experiments. Under our experimental conditions a slight shift to nicotine avoidance was measured that was weaker than the behavioral shift caused by varenicline treatment. The pretreatment did not result in a shift toward nicotine compartments. Similarly, learning in larval zebrafish appears to be limited as shown in certain learning paradigms (Valente et al., 2012; Roberts et al., 2013). Thus, mechanism needed for behavioral changes to occur in response to nicotine-pretreatment potentially could be lacking in the early larval stage of zebrafish.

## 5. Outlook

**Figure 7:**
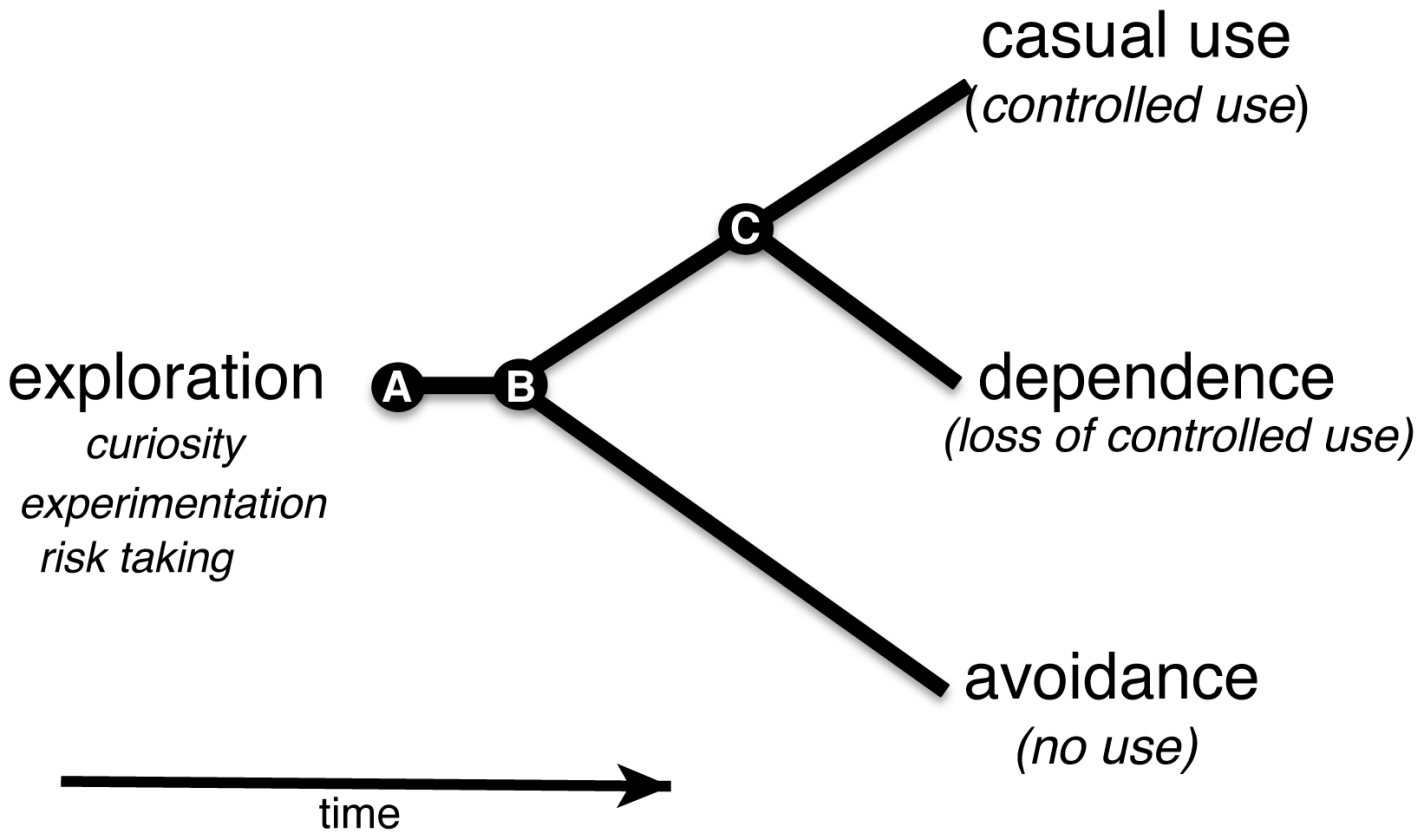
Shows a simplified behavioral cladogram of types of nicotine use behavior. The model is based on Jordan and Andersen (2017). **(A)** Use of nicotine is initiated early in development in an exploratory phase when curiosity, risk taking and experimentation drive behavior. The associated experience could lead to avoidance or continued nicotine use at the second transition point **(B)**. Continued use could put an organism on a path to a third transition point **(C)** at which loss of control over nicotine use could take place leading to nicotine dependence.

Studies of nicotine use in humans suggested that exploratory use of nicotine in early adolescence is an indicator of future nicotine-dependence (Jordan and Andersen, 2017). In the absence of an aversive response to nicotine in an exploratory phase, continued regular use of nicotine could result in a transition toward nicotine dependence (Figure 7; Smith et al., 2015). The model can be adopted for larval zebrafish with an exploratory phase in early development. The gradient maze test for the identification of nicotine-seekers and avoiders in the larval zebrafish model in combination with selected breeding or genome modifications could help to discover genetic risk factors that contribute directly to the transition from exploratory to regular (controlled) use of nicotine.

## Data availability statement

The original contributions presented in the study are included in the article/Supplementary material, further inquiries can be directed to the corresponding author.

## Ethics statement

The animal study was reviewed and approved by IACUC at DePauw University.

## Author contributions

All authors listed have made a substantial, direct, and intellectual contribution to the work and approved it for publication.

## Funding

This study was supported by grants from DePauw University (HS, AP, DH, AT, SK, KG, and KC), Arthur Vining Davis Foundations (HS), and the Buehler Family Foundation, A.C. Buehler and E. Buehler.

## Conflict of interest

The authors declare that the research was conducted in the absence of any commercial or financial relationships that could be construed as a potential conflict of interest.

## Publisher’s note

All claims expressed in this article are solely those of the authors and do not necessarily represent those of their affiliated organizations, or those of the publisher, the editors and the reviewers. Any product that may be evaluated in this article, or claim that may be made by its manufacturer, is not guaranteed or endorsed by the publisher.

## Footnotes

^1^https://www.cdc.gov/ – Smoking and Tobacco Use – Fast Facts and Fact Sheet, accessed 11-27-22.

^2^ Celltreat.com
