# Supplementary material for: Identification of nicotine-seeking and avoiding larval zebrafish using a new three-choice behavioral assay
Source: Front Mol Neurosci. 2023 Mar 30;16:1112927. doi: 10.3389/fnmol.2023.1112927 (PMC10098024; doi:10.3389/fnmol.2023.1112927)
Supplement: Supplementary file 1 [file Data_Sheet_1.PDF]

Supplemental material

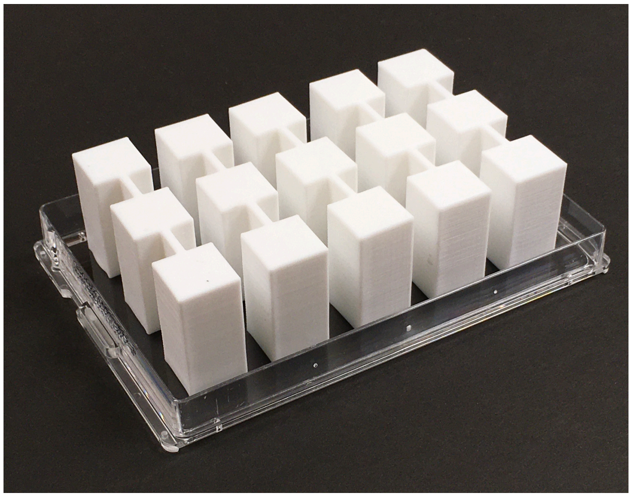

Figure S1 shows five gradient maze molds in a one-well plate before agarose solution was added.

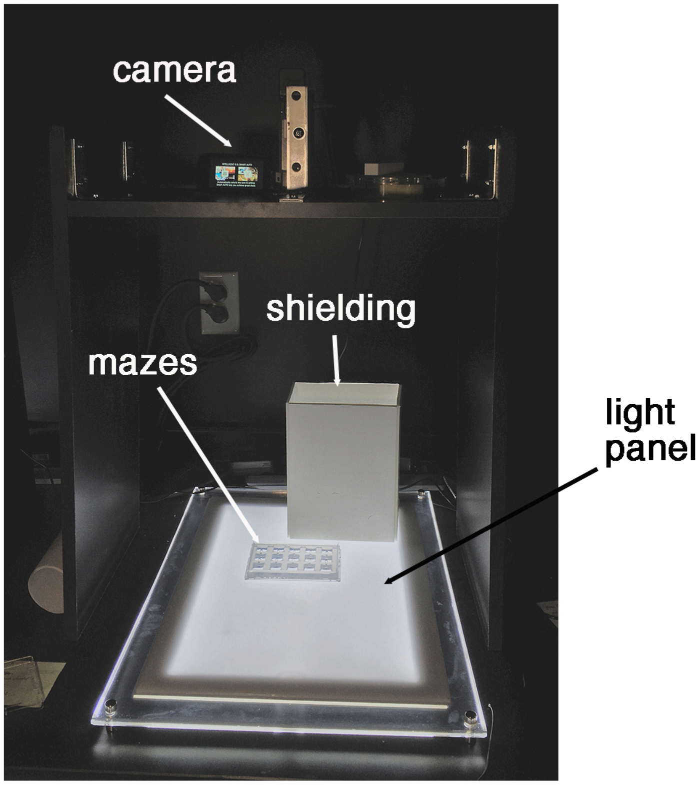

Figure S2 shows on of three identical behavioral setups that were used for experiments and video-recording.
